# A Radiomic Model for Gliomas Grade and Patient Survival Prediction

**DOI:** 10.3390/bioengineering12050450

**Published:** 2025-04-24

**Authors:** Ahmad Chaddad, Pingyue Jia, Yan Hu, Yousef Katib, Reem Kateb, Tareef Sahal Daqqaq

**Affiliations:** 1 Artificial Intelligence for Personalised Medicine, School of Artificial Intelligence, Guilin University of Electronic Technology, Guilin 541004, China; 2 Laboratory for Imagery, Vision and Artificial Intelligence, École de Technologie Supérieure (ETS), Montreal, QC H3C 1K3, Canada; 3 College of Medicine, Taibah University, Al Madinah 42361, Saudi Arabia; 4 College of Computer Science and Engineering, Jeddah University, Jeddah 23445, Saudi Arabia; 5 Cyber Security Department, College of Computer Science and Engineering, Taibah University, Al Madinah 42361, Saudi Arabia; 6 Department of Radiology, Prince Mohammed Bin Abdulaziz Hospital, Ministry of National Guard Health Affairs, Al Madinah 42324, Saudi Arabia

**Keywords:** radiomics, magnetic resonance imaging, brain tumor, classification, survival analysis

## Abstract

Brain tumors are among the most common malignant tumors of the central nervous system, with high mortality and recurrence rates. Radiomics extracts quantitative features from medical images, converting them into predictive biomarkers for tumor diagnosis, prognosis, and survival analysis. Despite the invasiveness and heterogeneity of brain tumors, even with timely treatment, the overall survival time or survival probability is not necessarily favorable. Therefore, accurate prediction of brain tumor grade and survival outcomes is important for personalized treatment. In this study, we propose a radiomic model for the non-invasive prediction of brain tumor grade and patient survival outcomes. We used four magnetic resonance imaging (MRI) sequences from 159 patients with glioma. Four classifiers were employed based on whether feature selection was applied. The features were derived from regions of interest identified and corrected either manually or automatically. The extreme gradient boosting (XGB) model with 3860 radiomic features achieved the highest classification performance, with an AUC of 98.20%, in distinguishing LGG from GBM images using manually corrected labels. Similarly, the Random Forest (RF) model exhibits the best discrimination between short-term and long-term survival groups with a *p*-value < 0.0003, a hazard ratio (HR) value of 3.24, and a 95% confidence interval (CI) of 1.63–4.43 based on the ICC features. The experimental findings demonstrate strong classification accuracy and effectively predict survival outcomes in glioma patients.

## 1. Introduction

Brain tumors pose significant challenges in medical diagnostics and treatment planning due to their varying pathologies and prognosis. Among these, gliomas are particularly notable, comprising approximately 80% of primary malignant brain tumors [1]. These tumors are classified into four grades according to their histological characteristics: Grade I: Non-invasive tumors, Grade II: Low-grade gliomas, Grade III: Anaplastic, intermediate grade gliomas (part of lower-grade gliomas, LGGs), and grade IV: Aggressive malignant tumors known as glioblastoma multiforme (GBM) [2]. In addition, other histological classifications include a variety of subtypes such as astrocytoma, oligoastrocytoma, and oligodendroglioma. Molecular pathology also plays an important role in the classification and prognosis of gliomas, with common molecular markers that include isocitrate dehydrogenase (IDH) 1/2, O6-methylguanine-DNA methyltransferase (MGMT) gene promoter methylation, loss of alpha thalassemia/mental retardation syndrome x-linked (ATRX), tumor protein (TP) 53 mutations, and complete deletion of both the short arm of chromosome 1 (1p) and the long arm of chromosome 19 (19q) [3]. The outcome for patients with gliomas depends on a combination of patient factors (e.g., age, performance status), treatment factors (e.g., extent of resection, adjuvant therapy options such as chemotherapy or chemoradiation), and tumor characteristics (e.g., types such as astrocytoma, oligodendroglioma, GBM, tumor size, location, histological grade, and molecular pathology) [4]. Accurate classification and understanding of these factors are critical for effective treatment and improved patient outcomes [5].

Recently, there has been a growing interest in applying AI models within the field of radiomics to clinical tasks [6,7,8]. Radiomics is a data-driven AI-based analysis method that extracts valuable information from imaging data by converting images into usable large-scale data. This approach has shown promise in the prediction of molecular genetic, tumor classification, differential diagnosis, and prognostic evaluations of gliomas [9]. Radiomic studies have extracted various radiomic features, including texture, first-order, and shape features, from patients with gliomas, to classify GBM from LGGs. In [10], features were selected using the elimination of recursive features with the RF algorithm. Five classifier models were constructed using these selected features: support vector machine (SVM), RF, gradient boost, naive Bayes, and AdaBoost classifiers. Among these models, the RF classifier performed the best, achieving an AUC of 0.81 for the test cohort. Another study retrospectively evaluated 212 patients with intracranial meningioma from two hospitals. The combined model achieved an AUC of 0.86 and an accuracy of 0.73 for meningioma grading, underscoring the potential value of radiomics along with clinical imaging features to predict the histological grade of meningioma. The combination of radiomics and clinical imaging features produced the highest AUC in the model [11]. In [12], three models based on least absolute shrinkage and selection operator (LASSO), SVM, and RF used MRI images with the T1-Weighted Imaging (T1WI), T2-Weighted Imaging (T2WI), Diffusion-Weighted Imaging (DWI), and T1-enhanced sequence to assess their diagnostic efficacy. The LASSO model showed the best performance, indicating that MRI images are effective in differentiating between gliosis and LGGs. In addition, radiomic models have shown promise in predicting patient survival related to brain tumors [13]. Several other studies have demonstrated the ability of radiomics to accurately predict gliomas survival rates at various stages [14]. For example, a study developed a prediction model using conventional multimodal preoperative MRI imaging features to classify glioma grades II, III, and IV, which helps in accurate diagnosis and optimized clinical management [15]. Furthermore, a fully automated radiomic model has been shown to facilitate the multiclass classification of single enhanced brain tumors, including GBM, central nervous system lymphoma, and metastases [16]. Another study used radiomic models and three-dimensional arterial spin labeling (3D-ASL) to distinguish high-grade gliomas (HGGs) from low-grade gliomas (LGGs), the multilayer perceptron model (MLP) demonstrating the best diagnostic performance [17].

In addition, predicting the survival of patients helps to plan treatment [18]. Survival analysis is prevalent in clinical trials and prognosis studies [19]. For example, LGG patients typically survive 10 years or more, outliving GBM patients [20]. When GBM recurs, even aggressive treatment yields a median survival of only 18–24 months [21]. The aggressiveness and heterogeneity of GBM cause wide variations in overall survival (OS), making survival analysis important for treatment planning. For survival analysis, regression models are used to predict OS or radiomics from preoperative images to classify survival as short- or long-term survival [22]. For example, an automated OS prediction system was developed using MRI images and additional data to categorize patients with GBM into short, medium and long-term survival groups, demonstrating higher predictive value in patients with glioma [23]. A study used 71 radiomic features from pre-operative MRI scans of 296 LGG patients to build a random survival forest model, which enhanced the accuracy of survival prediction for IDH status and clinical indicators. Adding radiomic features to the model significantly improved its accuracy [24]. The trained radiomics model effectively predicted OS in patients with both GBM and LGGs, and combining radiomics with clinical and genetic information further improved predictive performance [25]. Many studies show that tumor regions are closely related to OS and require manual segmentation [26]. However, manual segmentation is time-consuming, subjective, and non-repeatable, limiting clinical use. In [27], a post hoc method was introduced for OS prediction that does not require segmentation map annotations and uses only true OS time as a training signal. This method performed competitively against state-of-the-art pre-trained methods on the 2019 Multimodal Brain Tumor Segmentation Challenge (BraTS) dataset. Another investigation extracted location-based features of GBM to assess their independent and combined effect with radiomic features on OS prediction. The regression model indicated a significant independent impact of these features on OS, while the classifier model showed improved prediction accuracy when combining location-based and radiomic features [28]. In [29], a proposed framework for predicting OS in patients with GBM consists of two stages: first, brain tumor segmentation using the dynamic kernel network (DKNet) for enhanced segmentation; second, using a multilayer perceptron model to combine selected shape features, deep features, and clinical information to predict survival time. Despite advances in radiomic models to predict OS, there are limited studies that examine the effects of manual and automatic segmentation correction on OS in patients with glioma. With the progress of AI models in medical imaging, these challenges are being addressed. An automatic framework using multi-mode MRI was proposed for non-invasive OS prediction in patients with GBM [30]. This framework includes a 3D-Unet model to segment GBM into three sub-regions and a support vector regression (SVR) model to predict OS using imaging and clinical data. Specifically, AI with radiomics can quantify tumor regions, predict OS, and guide personalized treatment. However, more research is needed in this field.

This study proposes a radiomic pipeline for the classification of LGGs from GBM in patients with brain tumors and for conducting survival analysis. We combine features from four imaging modalities: T1WI, T2WI, T1-weighted Gadolinium-enhanced imaging (T1GD), and Fluid-Attenuated Inversion Recovery (FLAIR). These features are derived using two types of masks—manually corrected (Mask-A) and AI-model corrected (Mask-B). We apply two feature selection methods: the least absolute shrinkage and selection operator (LASSO) and the intraclass correlation coefficient (ICC), to identify significant and stable features. The impact of mask types and feature selection methods on classification and survival analysis is compared using four radiomic models based on RF, SVM, LR (logistic regression), and XGB. This study aims to illustrate the potential of AI and radiomic models to effectively classify glioma grades (LGGs and GBM) and predict patient survival outcomes. The main contributions of this study are as follows.

- We simulate four predictive models to classify between LGGs and GBM images using a manual and/or automatic corrected segmentation mask.
- We evaluate four predictive models in the survival analysis for the patient with brain tumor using LGG and GBM MRI images.
- We assess the impact of LASSO and ICC features in the classification of LGGs and GBM and the survival analysis.

The rest of this paper is organized as follows. Section 2 provides the details of our experiments. Section 3 presents the results using radiomic models. Section 4 discusses the findings. Finally, Section 5 summarizes the conclusions of this paper.

## 2. Method

**Figure 1:**
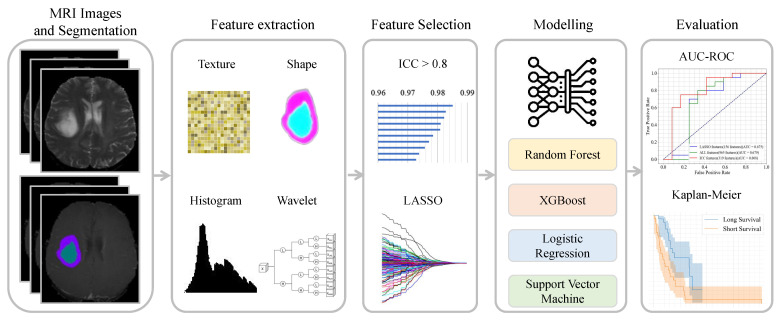
Flowchart of the radiomic pipeline. The input image, along with its corresponding masks (labels), is used to extract imaging features (e.g., texture, first-order, and shape features) using Pyradiomics. Important features based on ICC or LASSO and also the entire features are used to build the predictive models, which predict the grade of the tumor and the survival outcome of patients with glioma. The performance of the model is evaluated based on the test samples.

Figure 1 illustrates the radiomic pipeline that consists of the following: (1) the input images with the corresponding masks that are derived from the GLISTRboost segmentation mask after manual correction (known as A) or without manual correction (known as B), (2) the extraction of image features from the segmented region of interest (ROI), which converts the tumor image into quantitative features, (3) feature selection using the ICC and LASSO to select the dominant features, and (4) build a predictive model using the imaging features as input to predictive models to predict tumor grade and survival analysis. Finally, we used the data test set to measure the performance metrics of the models. Algorithm 1 shows the brief flow of this study.
**Algorithm 1** Pipeline of radiomics model.**Require:** Brain tumor MRI image *I*, segmentation masks $M_{A}$ and $M_{B}$ **Ensure:** Extracted features $F_{\mathrm{new}}$, trained models trained_models, performance metrics metrics
1:$segmented\text{\_}A\leftarrow I\cap M_{A}$2:$segmented\text{\_}B\leftarrow I\cap M_{B}$3:$F_{A}\leftarrow \mathrm{bad}\;\mathrm{hbox}\left(segmented\text{\_}A\right)$4:$F_{B}\leftarrow \mathrm{bad}\;\mathrm{hbox}\left(segmented\text{\_}B\right)$5:$stable\text{\_}features\leftarrow \mathrm{bad}\;\mathrm{hbox}(F_{A},F_{B})$6:$important\text{\_}features\leftarrow \mathrm{LASSO}(F_{A},F_{B})$7:$F_{\mathrm{new}}\leftarrow \mathrm{select}\left(stable\text{\_}features,important\text{\_}features\right)$8:$trained\text{\_}models\leftarrow \mathrm{bad}\;\mathrm{hbox}(models,F_{\mathrm{new}})$9:metrics←bad hbox(trained_models,Test_samples)▹ Segment image using mask $M_{A}$ ▹ Segment image using mask $M_{B}$ ▹ Extract features from the region of $M_{A}$ ▹ Extract features from the region of $M_{B}$ ▹ Apply ICC ▹ Apply LASSO regression ▹ Select stable and important features ▹ Train models using selected features ▹ Evaluate models using the test samples


### 2.1. Dataset Description

This study used two publicly accessible datasets (BraTS-TCGA-LGG and BraTS-TCGA-GBM [31]) from the Cancer Imaging Archive (TCIA) data platform, with the patient data consisting of MRI images. These MRI images underwent a series of pre-processing steps, including repositioning in the left posterior superior coordinate system (LPS), alignment with a unified anatomical template, resampling to a voxel resolution of 1 × 1 × 1 mm, and skull stripping [32]. We used masks derived from an implementation of glioma image segmentation and alignment using the GLISTRboost software [31]. The data consist of two types of segmentation mask: (1) With the suffix “ManuallyCorrected.nii.gz”, it represents Mask A. and (2) With the suffix “_GlistrBoost.nii.gz”, it represents Mask B. In this study, these two masks (A and B) were considered.

### 2.2. Patients and Data Cleaning

Eight patients were excluded from further analysis due to the absence of GLISTRboost segmentation masks or manually corrected segmentation masks. The demographic data of the patients included in the study comprised a total of 159 patients after data cleaning. Specifically, 97 patients with GBM and 62 patients with LGGs. The description and distribution of patient details can be found in Table 1.

### 2.3. Feature Extraction

Radiomic features were calculated using the PyRadiomics software package [33]. These features were carefully selected to ensure clinical relevance and computational accuracy. They are categorized into several groups: (1) intensity features (*n* = 3), which include raw intensity values and differences across modalities; (2) volumetric feature (*n* = 2), describing the volume properties of the tumor regions; (3) morphologic features (*n* = 14), characterizing the shape and form of the tumor; (4) histogram-based features (*n* = 18), derived from the distribution of intensity values within the tumor region; and (5) textural features based on gray-level co-occurrence matrix (GLCM, *n* = 22), gray-level run-length matrix (GLRLM, *n* = 16), gray-level size zone matrix (GLSZM, *n* = 16), and gray-level dependence-matrix (GLDM, *n* = 14) [34,35]. These five categories provide a total of 105 features.

After we applied wavelet transformation and the Laplacian Gaussian (LoG) filter, we calculated the 18 histogram + 68 texture features through eight wavelet decomposition directions (i.e., LLH, LHL, LHH, HLL, HHL, HHL, HHH, and LLL) and two LoG filters (σ = 1 mm and 3 mm). Based on each direction, we obtained 86 features for each of these 10 filters (wavelet = 86 × 8, LoG = 86 × 2), to combine in a total of 860 features. So far, we combined these features (*n* = 105 + 860 features) to obtain a total of 965 features derived from each of the four MRI sequences.

### 2.4. Feature Selection

We used the ICC and LASSO methods to select the most significant features from the training datasets, considering masks A and B.

#### 2.4.1. Interclass Correlation Coefficient-ICC

Using the “psych” package in the R language, the ICC was calculated for each feature. Features with an ICC greater than 0.80 were considered reliable, while those with an ICC of 0.80 or less were deemed unreliable. This cut-off, based on established guidelines [36], signifies excellent reliability and ensures that the selected features exhibit high reproducibility and consistency, which is important for radiomic analysis. This threshold ensures that the selected features exhibit high reproducibility and consistency under various conditions, which is important for robust radiomic analysis.

For this study, 3860 features were extracted individually. The two feature matrices, $T_{1}$ and $T_{2}$, were then merged column by column to obtain a new matrix $T_{12}$. The ICC(2,1) of the corresponding columns of $T_{1}$ and $T_{2}$ was calculated for each radiomic feature *i*, where i=1,2,…,3860. In this context, *i* represents the index of each feature being analyzed, with a total of 3860 features across all the extracted radiomic features. Features with an ICC greater than 0.8 were considered reliable. Consequently, 1507 features were selected for further classification analysis between LGGs and GBM, as well as for survival analysis, using either mask A or mask B.

#### 2.4.2. LASSO

It adds a penalty to the error term based on the absolute value of the magnitudes of the coefficients, reducing some coefficients to zero and simplifying the model to avoid overfitting [37]. The LASSO optimization problem can be defined as follows:

(1) $$\begin{array}{cc} \hat{\beta } & =\arg\min_{\beta }\left(\sum_{i=1}^{n}{\left(y_{i}\;-\;x_{i}^{T}\beta \right)}^{2}+\lambda \sum_{j=1}^{p}\left|\beta_{j}\right|\right) \end{array}$$

where $\hat{\beta }$ is the vector of estimated regression coefficients, representing the importance or contribution of each radiomic feature in predicting the outcome. $y_{i}$ is the observed response for the *i*-th subject (e.g., survival). $x_{i}^{T}$ is the feature vector for the *i*-th subject, which contains the radiomic features. β represents the model coefficients that are estimated through the optimization process. These coefficients indicate the weight or impact each radiomic feature has on the prediction. $\beta_{j}$ is the *j*-th coefficient in the vector β, corresponding to the weight of the *j*-the radiomic feature used as input in the model. *n* is the number of samples in the dataset being analyzed. *p* is the number of radiomic features considered as predictors for the model. λ is the regularization parameter that controls the strength of the penalty term. We set the range of λ in the LASSO method from $10^{-10}$ to $10^{-1}$. Using 5-fold cross-validation, we determine the optimal λ by identifying the one with the lowest validation error. Subsequently, we select features based on the non-zero coefficients of the model at the chosen λ.

Specifically, increasing the value of λ causes more coefficients to shrink to zero, thereby simplifying the model. Using 5-fold cross-validated LASSO on training datasets for feature selection, we obtained 290 features (mask A) and 286 features (mask B) for classification between LGGs and GBM, respectively. We selected a total of 1416 features based on mask A and 3856 features based on mask B for survival analysis.

### 2.5. Experimental Environment Details and Modeling

We used Python 3.8 for feature extraction. The versions for R and pyRadiomics are 4.4.1 and 3.1.0, respectively. Our experimental setup was operated on a Windows 11 platform, powered by an Intel Core i9-13900KF CPU and a Nvidia RTX 4090 GPU.

Using the features as input for the predictive models, we build these models using the training datasets (*n* = 111 samples, 70%) and subsequently evaluate them with the test datasets (*n* = 48 samples, 30%). Another split for training and testing could also be used. However, we considered this splitting to represent the imbalanced sample between the GBM and LGG samples.

We classify LGG from GBM images using RF, LR, SVM, and XGB classifier models.

Specifically, RF performs classification and prediction using an ensemble of decision trees, which enhances prediction accuracy and avoids overfitting by averaging or majority voting. This study uses an RF model with 300 trees, where randomness in sampling and feature selection reduces variance and improves overall performance.

In LR, the probability of the outcome is modeled using a logistic function and various regularization techniques like LASSO (L1), Ridge (L2), and Elastic-Net are used to handle many types of regression problems. In this study, the LR model uses L2 regularization with a regularization strength C of 1, “liblinear” as the solver, and limits the maximum number of iterations to 100.

SVM aims to maximize the intervals between classes by mapping the data to high-dimensional feature spaces and using hyperplanes for division. In this study, we used the linear kernel implementation with regulator C = 1, and the class_weight parameter was set to None.

XGB is an ensemble method known for its efficiency and accuracy. It combines multiple predictors, typically decision trees, to improve model performance by iteratively correcting for errors. XGB effectively handles complex data relationships, reduces overfitting, and offers faster training compared to traditional tree-based algorithms. This study uses the default parameter settings of the XGBClassifier function from XGB library.

### 2.6. Performance Evaluation

We evaluate the performance of the classifier models in test datasets using the following metrics: *Accuracy*, *Precision*, *Sensitivity*, *Specificity*, *F-score*, the confusion matrix, the area under the ROC curve (AUC), and decision curve analysis (DCA). The assessment is based on various indicators derived from true positives (TP) and true negatives (TN). Accurate identification of brain tumors is closely related to the proportions of true positives and true negatives, while the rates of false positives (FP) and false negatives (FN) highlight the inaccuracies in tumor detection. *Accuracy*, *Precision*, *Sensitivity*, *Specificity*, and *F-score* can be formulated as follows:

(2) $$\begin{array}{cc} Accuracy & =\frac{TP+TN}{TP+TN+FP+FN} \end{array}$$

(3) $$\begin{array}{cc} Precision & =\frac{TP}{TP+FP} \end{array}$$

(4) $$\begin{array}{cc} Sensitivity & =\frac{TP}{TP+FN} \end{array}$$

(5) $$\begin{array}{cc} Specificity & =\frac{TN}{TN+FP} \end{array}$$

(6) $$\begin{array}{cc} F-score(F1) & =2\times \frac{TP}{2TP+FP+FN} \end{array}$$

Specifically, the confusion matrix identifies the correctly and incorrectly classified samples in each class. The AUC-ROC is an important indicator for evaluating the performance of a classifier model. In addition, the AUC is computed to evaluate the performance of a classification model; for example, ranging from 0 to 1. A value closer to 1 indicates better performance. DCA is used to assess the clinical applicability of the model by analyzing the net benefit at threshold probabilities [38]. Despite the confusion matrix and the ROC curve that illustrate the performance metrics of the model, DCA evaluates their clinical applicability. Combining these methods offers a comprehensive view of model performance and potential utility in clinical practice.

### 2.7. Survival Analysis

For the survival analysis, we used 111 samples for training and 48 samples for testing. Patients were classified into short and long survival groups based on the median survival time of 516 days among the 159 patients to balance the distribution between short- and long-term survival groups. Since the class samples are balanced, another split of training and testing could also be used. We evaluated the survival analysis of patients with LGGs and GBM tumors using four predictive models.

The Kaplan–Meier estimator was used to visualize the long and short survival curves in the test set. Differences between survival curves were evaluated using the logarithmic rank test (log rank) with a significance threshold of 0.05 (i.e., *p* < 0.05 indicates significantly distinguishable survival curves). Kaplan–Meier curves were generated using the Lifelines library version 0.28.0 in Python.

We considered patient death an event (OS = 1) and patient survival or loss to follow-up as event censoring (OS = 0). For two patients with unknown outcomes, survival days were set at the median survival days of all patients (516 days).

To further assess the survival differences between the two groups, a Cox Proportional Hazards (CPH) model was used to evaluate the effect of covariates on survival time. In this study, the CPH model was fitted to the test dataset, providing a hazard ratio (HR) that indicates the relative risk of the short survival group compared to the long survival group, along with a confidence interval 95%. HR was calculated as the exponentiated coefficient ($e^{\beta }$) of the fitted CPH model, where β represents the estimated log hazard difference between the two groups. The results of the CPH model complement the findings of the Kaplan–Meier and log rank tests, offering additional information on the prognostic impact of group assignments on survival outcomes.

## 3. Result

**Figure 2:**
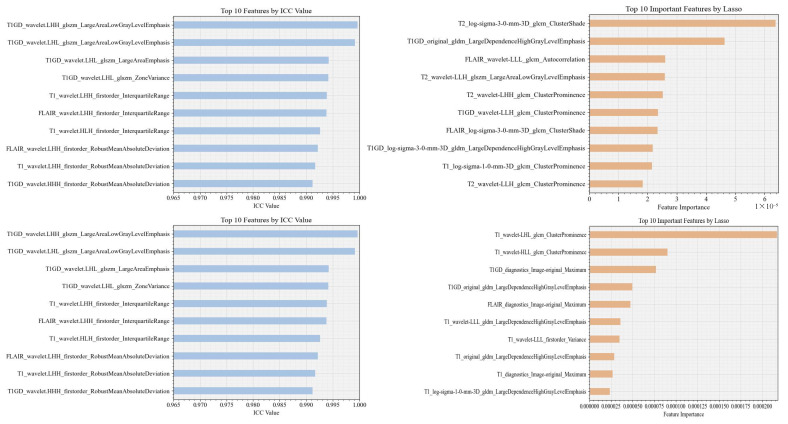
Top 10 important features identified using ICC (**left**) and LASSO (**right**) models in classification. The first and second rows represent masks A and B, respectively.

**Dominant features:** Figure 2 illustrates the top-ranked features based on their importance in classifying LGGs from GBM. For example, the feature with the highest importance according to ICC is identified as *wavelet LHH GLSZM Large Area Low Gray Level Emphasis* from T1GD with the application of mask A and B. However, when using LASSO, the leading feature is *Log-Sigma-3-0-mm-3D GLCM Cluster Shade* from T2WI with mask A. For mask B, the leading feature is *Wavelet LHL GLCM Cluster Prominence* from T1WI. As illustrated, the key features selected using the ICC are consistent in both mask A and mask B, demonstrating the stability of these features. However, the LASSO method shows different important selected features between masks A and B.

**Figure 3:**
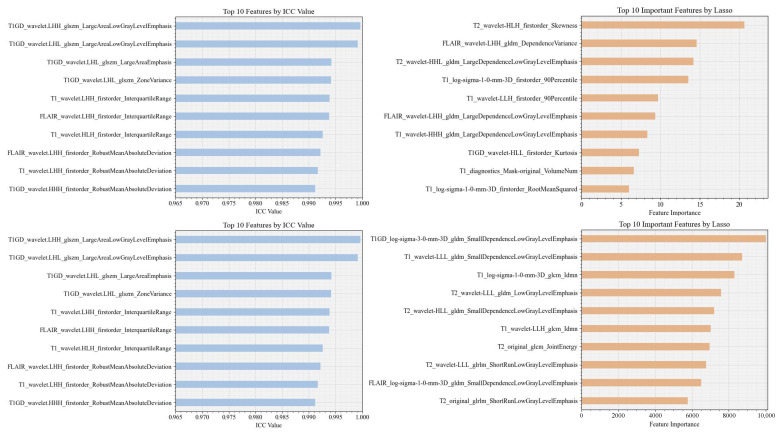
Top 10 important features identified in survival analysis using ICC (**left**) and LASSO (**right**) models. The first and second rows represent masks A and B, respectively.

In the same context, Figure 3 illustrates the top-ranked features based on their importance in classifying short- and long-term survival. For example, with the LASSO method and mask A, the leading feature is *Wavelet HLH first order Skewness* from T2WI, while *Log Sigma-3-0-mm-3D GLDM Small Dependence Low Gray Level Emphasis* from T1GD is the most important feature with mask B. These results highlight the varying significance of imaging features for model prediction depending on the feature selection method, the segmentation masks used, and the tasks.

**Figure 4:**
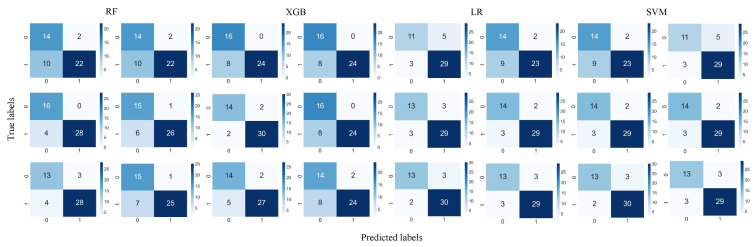
Confusion matrices for four classifier models using sets of features. LGGs and GBM are labeled as 0 and 1, respectively. The rows from top to bottom represent features selected by ICC, ALL features, and LASSO, respectively. Left denotes manually corrected segmentation labels, while right denotes a computer-assisted segmentation mask implemented by GLISTRboost without manual correction.

**Figure 5:**
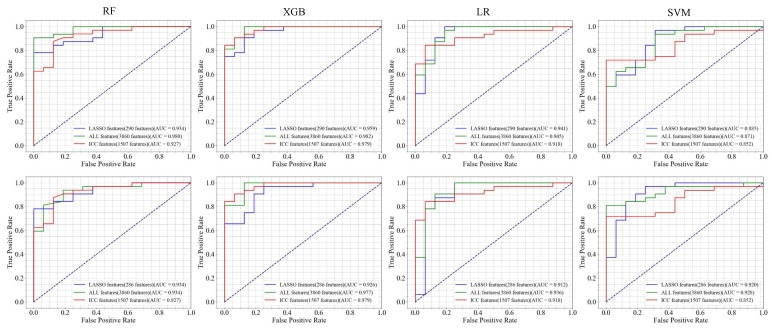
ROC curves for four classifier models using different features. The columns from one to four represent the RF, XGB, LR, and SVM classifier models. The top row represents manually corrected segmentation labels (mask A), while the bottom row represents a computer-assisted segmentation (mask B) by GLISTRboost without manual correction.

**LGG versus GBM:** Table 2 reports the results of the classification between LGGs and GBM using the full and selected features (LASSO and ICC) with the RF, SVM, LR, and XGB classifier under two mask conditions (mask A and mask B). In addition, Figure 4 and Figure 5 show the confusion matrix and ROC curves of the classification between LGGs and GBM.

**Figure 6:**
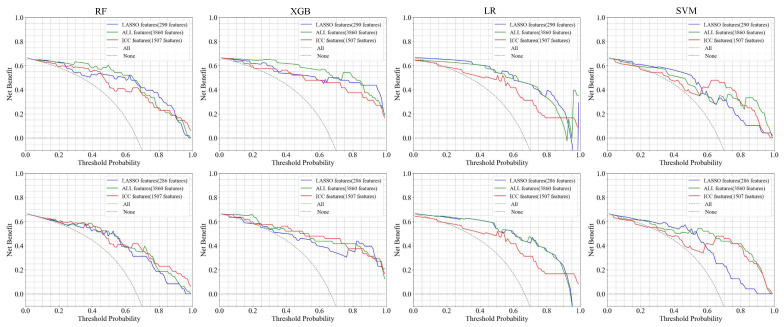
Decision curve analysis (DCA) for four classifier models employing various features. The columns from one to four represent the RF, XGB, LR, and SVM classifier models. The top and bottom rows correspond to mask A and mask B, respectively.

Based on the LASSO feature, the LR and SVM model with mask A provides the highest accuracy of 89.58%. The AUC of XGB using mask A/B is 0.959/0.926, showing a high model prediction ability. In addition, the F-score for the LR model using mask A can reach 92.31%. The confusion matrices reveal that mask B predictions exhibit a class imbalance, while mask A shows a more stable error distribution. Using ICC features, the LR and XGB models achieved a superior accuracy of 83.33% for masks A and B. The XGB model also showed the highest AUC of 0.979 for both masks. When using all features, the RF and XGB models with mask A reached the highest accuracy of 91.67%, while the XGB model achieved the top AUC of 0.982, outperforming in three feature groups: LASSO, ALL feature, and ICC. Overall, XGB generally provides a higher AUC and other performance metrics, demonstrating good generalization with variations depending on the mask. In addition, the combination of ICC and LASSO features provides consistent results, in the same range of using ICC and LASSO individually. In addition, Figure 6 illustrates the DCA curves. We find that, for the XGB model, the net benefit of masks A and B is higher than for the RF, LR, and SVM models.

Table 3 reports the results of the testing using five-fold cross-validation for the classification of brain tumors. For LASSO features, the XGB model achieved the highest accuracy of 83.71% using mask A. The XGB model using mask B achieved the highest sensitivity of 90.84%.

When all features were used, the RF model obtains the highest accuracy of mask A, while the model with the highest accuracy and precision using mask B was XGB, which were 88.69% and 89.92%, respectively. However, in the remaining models, the accuracy and precision of mask A were higher than those of mask B, indicating that mask A was more stable.

Using ICC features, the two masks used by XGB achieved an accuracy of 88.04% and 87.44%, respectively, which were higher than those of SVM and LR. When combining ICC and LASSO features, the accuracy of the RF model using mask A reaches 88.02%, and also reaches the highest F-score of 90.47%, consistent with the results in Table 2.

**Figure 7:**
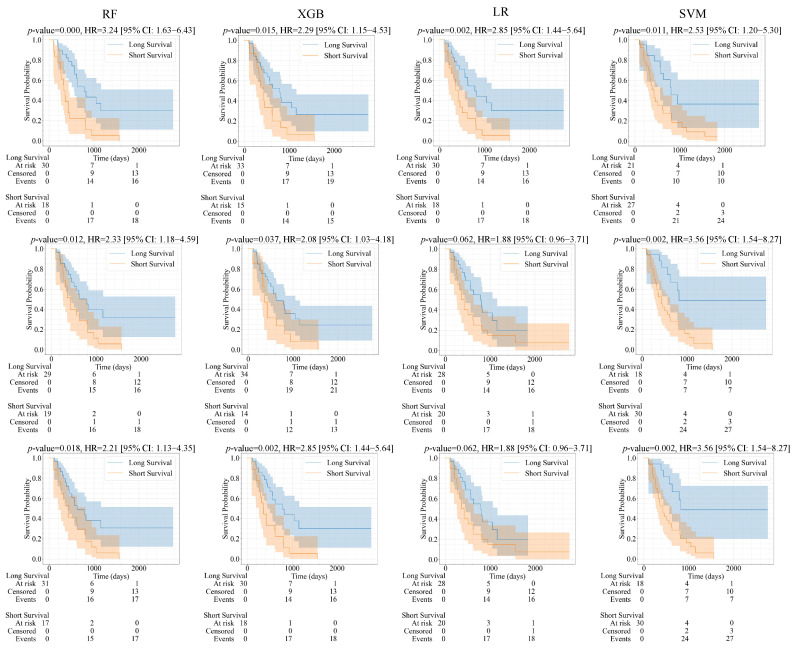
Kaplan–Meier survival curves for predicted short and long survival groups. Columns (left to right): RF, SVM, LR, and XGB. Rows (top to bottom): ICC, ALL features, and LASSO. Features are derived from segmentation of mask A.

**Figure 8:**
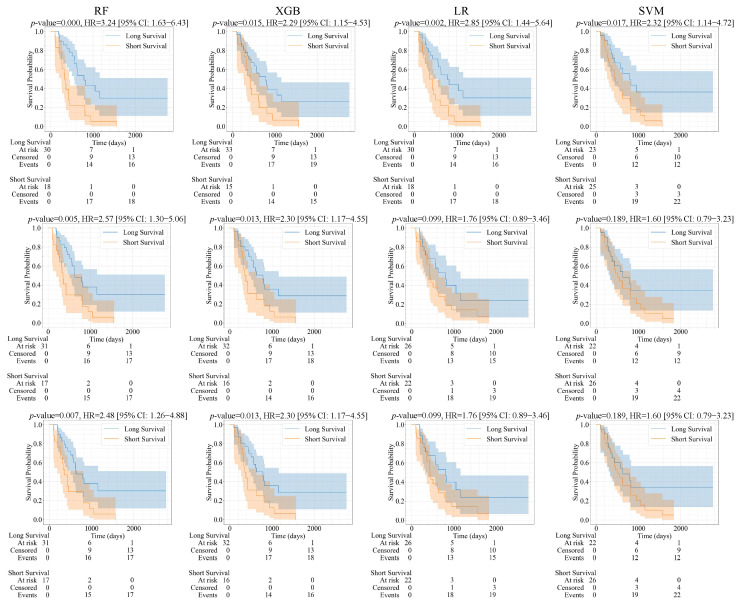
Kaplan–Meier survival curves showing predicted short and long survival groups. The columns (left to right) represent four classifier models (RF, SVM, LR, XGB). The rows (top to bottom) represent three feature sets (ICC, ALL features, LASSO) derived from segmentation of mask B.

**Survival analysis:** For the survival analysis, we also used 111 samples for training and 48 samples for testing, with the same patient ID (index) as brain tumor classification. Patients were classified into short and long survival groups based on the median survival outcome of 516 days. The predicted samples (test set) and their corresponding survival data were used to apply the Kaplan–Meier estimator and log-rank significance test to determine the significance of the predicted survival groups. As illustrated in Figure 7 and Figure 8, the Kaplan–Meier survival curves were generated using the predicted groups of test samples using the full feature sets, LASSO, and ICC features, respectively. There are four models and three sets of radiomic features. Therefore, the number is 12 models in total. Specifically, differences between survival curves were assessed using the log-rank test with a significance threshold of 0.05 (i.e., *p* < 0.05 indicates significantly distinguishable survival curves). Kaplan–Meier curves were generated using the Lifelines library version 0.28.0 in Python.

We considered patient death as an event (OS = 1), and patient survival or loss to follow-up as event censoring (OS = 0). For two patients with unknown outcomes, the survival days were set to the median survival days of all patients (516 days).

In Figure 7 and Table 4, 10 predictive models that considered mask A are significantly associated with the survival outcome of the patient with glioma with *p* <0.05 (i.e., LASSO-RF, LASSO-SVM, LASSO-XGB, ALL-RF, ALL-SVM, ALL- XGB, ICC -RF, ICC -SVM, ICC-LR, ICC- XGB). We observed that the metrics [p, HR, 95% CI] of ALL features (and LASSO)-SVM [0.002, 3.56, 1.54–8.27], achieve the highest significant differentiation between short and long survival. For example, ICC-RF achieved the highest C-index with a value of 0.719, followed by combined ICC and LASSO -RF with a value of 0.701. While, ICC-XGB performed best with a Brier score of 0.085. In Figure 8 and Table 4, eight predictive models considering mask B are significantly associated with glioma patient survival *p* < 0.05 (i.e., LASSO-RF, LASSO-XGB, ALL-RF, ALL-XGB, ICC-RF, ICC-SVM, ICC-LR, ICC-XGB). ICC-RF [0.0003, 3.24, 1.63–4.43] achieves the best differentiation between the short and long survival groups. In addition, ICC-RF also achieved the highest C-index, with a value of 0.719, and one more C-index greater than 0.7 than mask A. Meanwhile, the Brier score was the best for combined ICC and LASSO-XGB, with a value of 0.073. So far, whether using LASSO, all features, or ICC, most of the radiomics models have yielded significant results. This demonstrates the feasibility of imaging features being associated with survival outcomes.

### Ablation Study

**Figure 9:**
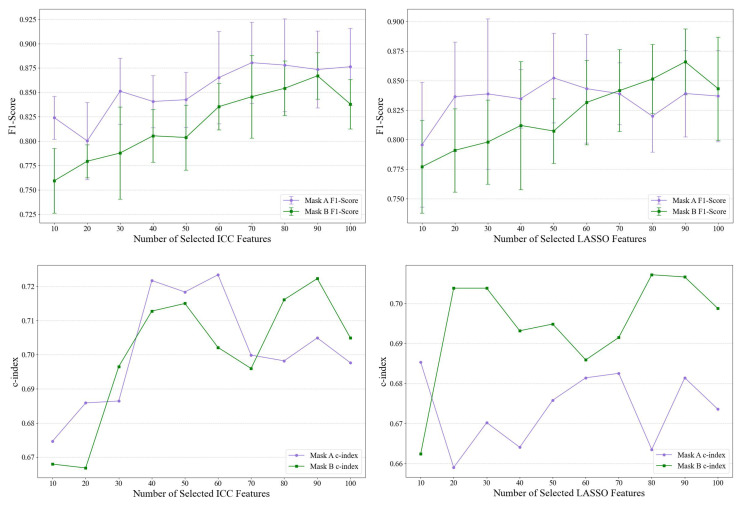
Random Forest models based on two masks, A and B: (**Top**) F-score performance for classifying LGGs and GBM based on 5-fold cross-validation. (**Bottom**) C-index representing the predicted survival time of patients (LGG+GBM) using the top dominant selected features based on ICC (**left**) and LASSO (**right**).

Based on the results of Table 3 and Table 4, we chose the RF model to perform the impact of ICC and LASSO on feature selection for the classification of brain tumors and the prediction of survival. Specifically, we selected the dominant features (top 10 to 100) based on ICC and LASSO, as shown in Figure 9. We considered the F-score based on 5-fold cross-validation for the classification of brain tumors and the C-index for survival analysis. So far, the F-score based on the top ICC and LASSO features ranges from 0.725 to 0.88, with mask A showing better performance in ICC. For survival analysis, RF models provide a C-index range value of 0.65–0.72. These results are consistent with previous results reported in Table 3 and Table 4.

## 4. Discussion

Recent studies in radiomics have significantly improved the precision of brain tumor characterization [39]. The integration of quantitative imaging features extracted from MRI images with ML algorithms is a remarkable approach to diagnosing and predicting the prognoses of brain tumors [19], particularly LGGs and GBM [2]. For example, in [2], they explore the relationship between deep radiomic features (DRFs) and immune cell markers to predict the survival of patients with glioma. Our study focuses on glioma classification (LGGs vs. GBM) and survival prediction using radiomic models, focusing on feature selection and segmentation mask impacts to enhance model accuracy.

Specifically, this study compared the effects of radiomic features considering both the entire set of features and the selected features (ICC and LASSO) on model performance. Although feature selection has been widely used in radiomics research, direct comparative studies applying these methods to manually adjusted segmentation masks (mask A) and automatically adjusted masks (mask B) are limited in the existing literature. The results of this study demonstrated the effects of feature selection on the prediction model, regardless of whether the labels were segmented and corrected manually or automatically (Figure 4).

In addition, the choice of the feature selection method (e.g., LASSO, ICC) widely affects the importance ranking of radiomic features (Figure 2). For example, the top feature identified by ICC (*wavelet LHH GLSZM Large Area Low Gray Level Emphasis* from T1GD) was consistent across both segmentation masks A and B, highlighting these feature’s stability. However, the LASSO method identified *Log-Sigma-3-0-mm-3D GLCM Cluster Shade* from T2WI / *Wavelet LHL GLCM Cluster Prominence* from T1WI as the dominant feature, in masks A/B. These features reflect the typical textural and intensity patterns associated with each tumor type, enabling the LASSO method to successfully capture the heterogeneity for classifying LGG from GBM.

In survival analysis, most radiomic models showed significant associations with survival outcomes, further demonstrating the potential of radiomic signatures to predict survival in patients with gliomas. For example, the ICC-RF model performed the most prominently among all models, with a significant *p*-value (0.0003). This result may be attributed to the fact that the features selected for ICC were more focused on survival-related imaging features, which improved the predictive power of the model.

In addition, the performance of the model was sensitive to the chosen mask. This sensitivity is due to reasons like minor interactions among selected features, segmentation specific biases that modify feature representation, and the nature of radiomic features. Even small changes in segmentation can notably shift feature distribution and model behavior.

However, our study has limitations. We did not compare our radiomic models with deep learning (DL) models, such as convolutional neural networks, nor did we incorporate molecular data such as genomic and proteomic information, due to computational constraints and focus limitations. Future work will address these limitations by incorporating DL models and integrating multi-dimensional features to enhance model robustness. Furthermore, future work will consider additional survival classes, more closely aligned with the clinical significance of brain tumors (e.g., the first group < second group: 3–6 months > third group). More datasets will also be used for testing to further validate our findings. Furthermore, although this study provides information on model performance, future research could address clinical validation and integration to ensure the applicability of the model in a hospital setting.

## 5. Conclusions

This study has demonstrated the effective use of a radiomic pipeline for the classification of LGGs and GBM, as well as for the prediction of overall survival in patients with glioma. Using robust feature selection methods such as LASSO and ICC, we identified the dominant features for classification tasks. Specifically, this study links radiomic analysis with clinical practice by offering tools to improve glioma management. Furthermore, this study demonstrates the advantages and limitations of common ML models in processing radiomics data, providing an important reference for future research.

## Figures and Tables

**Table 1:** Patient characteristics in the study.

GBM	
Number of patients	97
Number of segments (lesions)	194
Age (years)	
Median (Range)	56.5 (18–84)
Gender	
Male	58
Female	38
Unknown	1
Os.time(days)	
Median (Range)	424.5 (5–2768)
LGG	
Number of patients	62
Number of segments (lesions)	124
Age (years)	
Median (Range)	43.5 (20–74)
Gender	
Male	26
Female	36
Os.time(days)	
Median (Range)	788 (3–4752)

**Table 2:** Performance metrics (%) of machine learning models for brain tumor classification.

Feature (A/B)	Model	Accuracy	Sensitivity	Specificity	Precision	F-Score
LASSO features (290/286 features)	RF	85.42/83.33	87.50/78.13	81.25/93.75	90.32/96.15	88.89/86.21
	SVM	89.58/87.50	93.75/90.63	81.25/81.25	90.91/90.63	88.52/82.76
	LR	89.58/87.50	93.75/90.63	81.25/81.25	90.91/90.63	92.31/90.63
	XGB	85.42/79.17	84.38/75.00	87.50/87.50	93.10/92.30	88.52/82.76
ALL features (3860/3860 features)	RF	91.67/85.42	87.50/81.25	100.00/93.75	100.00/96.30	93.33/88.14
	SVM	87.50/89.58	90.63/90.63	81.25/87.50	90.63/93.55	90.63/92.06
	LR	87.50/89.58	90.63/90.63	81.25/87.50	86.67/92.86	90.63/93.55
	XGB	91.67/83.33	93.75/75.00	87.50/100	93.75/100	93.75/85.71
ICC features (1507/1507 features)	RF	75.00/75.00	68.75/68.75	87.50/87.50	91.67/91.67	78.57/78.57
	SVM	77.08/83.33	71.88/90.63	87.50/68.75	92.00/85.29	80.70/87.88
	LR	83.33/83.33	90.63/90.63	68.75/68.75	85.29/85.29	87.88/87.88
	XGB	83.33/83.33	75.00/75.00	100.00/100.00	100.00/100.00	85.71/85.71
Combined ICC and LASSO (1715/1709 features)	RF	89.58/81.25	84.38/75.00	100.00/93.75	100.00/96.00	91.53/84.21
	SVM	85.42/85.42	84.38/78.13	87.50/100.00	93.10/100.00	88.52/87. 72
	LR	85.42/85.42	84.38/78.13	87.50/100.00	93.10/100.00	88.52/87.72
	XGB	89.58/83.33	90.63/78.13	87.50/93.75	93.55/96.15	92.06/86.21

A/B, A uses manually corrected segmentation labels and B uses a computer-assisted segmentation mask implemented by GLISTRboost but not manually corrected.

**Table 3:** Performance metrics (average ± standard deviation) of ML models for brain tumor classification based on 5-fold cross-validation.

Feature (A/B)	Model	Accuracy	Sensitivity	Specificity	Precision	F-Score
	RF	83.08 ± 7.23/81.13 ± 3.43	86.79 ± 7.98/89.79 ± 4.36	77.44 ± 9.42/67.82 ± 8.15	85.87 ± 5.46/81.52 ± 3.82	86.19 ± 5.95/85.32 ± 2.47
LASSO features	SVM	77.98 ± 4.43/77.98 ± 6.56	85.68 ± 5.71/86.68 ± 3.76	66.03 ± 4.01/64.36 ± 13.85	79.79 ± 1.92/79.64 ± 6.44	82.58 ± 3.48/82.90 ± 4.45
(290/286 features)	LR	77.98 ± 1.99/83.63 ± 3.73	87.68 ± 3.85/88.68 ± 3.81	62.82 ± 8.67/76.03 ± 9.56	78.94 ± 3.25/85.47 ± 5.47	82.94 ± 1.12/86.88 ± 2.83
	XGB	83.71 ± 9.31/82.98 ± 6.84	88.84 ± 8.56/90.84 ± 7.48	75.77 ± 11.65/70.77 ± 8.76	85.23 ± 6.94/83.07 ± 4.51	86.95 ± 7.43/86.69 ± 5.30
	RF	87.42 ± 1.98/86.79 ± 4.14	91.74 ± 2.57/91.74 ± 4.20	80.51 ± 6.95/78.85 ± 6.77	88.31 ± 2.86/87.35 ± 3.76	89.93 ± 1.30/89.45 ± 3.45
ALL features	SVM	85.52 ± 4.27/80.54 ± 4.89	88.57 ± 4.06/84.47 ± 4.84	80.51 ± 10.73/74.23 ± 9.05	88.27 ± 6.68/83.91 ± 5.19	88.22 ± 3.40/84.09 ± 4.11
(3860/3860 features)	LR	84.90 ± 3.67/84.29 ± 2.71	86.47 ± 6.44/87.63 ± 2.49	82.18 ± 3.64/79.10 ± 3.52	88.39 ± 1.92/86.74 ± 2.47	87.31 ± 3.68/87.17 ± 2.31
	XGB	86.81 ± 2.24/88.69 ± 4.21	92.74 ± 2.67/91.74 ± 7.13	77.31 ± 6.46/83.85 ± 5.31	86.69 ± 2.77/89.92 ± 2.87	89.56 ± 1.76/90.66 ± 3.99
	RF	86.77 ± 4.21/86.79 ± 3.05	91.68 ± 4.29/90.68 ± 6.16	79.10 ± 6.34/80.51 ± 4.53	87.26 ± 3.94/88.06 ± 1.79	89.37 ± 3.60/89.22 ± 3.07
ICC features	SVM	85.51 ± 3.90/84.88 ± 3.77	85.42 ± 7.12/92.68 ± 7.89	85.26 ± 6.51/72.56 ± 12.54	90.43 ± 3.49/84.80 ± 5.99	87.63 ± 3.80/88.12 ± 3.10
(1507/1507 features)	LR	84.23 ± 4.62/86.11 ± 4.92	84.37 ± 7.59/87.53 ± 5.48	83.72 ± 5.49/83.59 ± 9.37	89.21 ± 3.28/89.73 ± 5.17	86.52 ± 4.41/88.47 ± 4.01
	XGB	88.04 ± 1.30/87.44 ± 3.37	93.79 ± 2.16/90.74 ± 7.64	78.97 ± 4.16/82.31 ± 5.94	87.55 ± 2.02/89.05 ± 3.17	90.53 ± 1.12/89.63 ± 3.42
	RF	88.02 ± 3.75/87.42 ± 1.98	92.74 ± 2.67/91.74 ± 5.36	80.51 ± 8.72/80.26 ± 11.57	88.42 ± 4.30/88.76 ± 5.26	90.47 ± 2.83/89.93 ± 1.24
Combined ICC and LASSO	SVM	86.15 ± 2.58/85.52 ± 1.62	87.53 ± 5.48/89.63 ± 7.48	83.46 ± 11.88/78.59 ± 13.79	90.27 ± 6.22/87.93 ± 6.03	88.54 ± 2.02/88.27 ± 1.41
(1715/1709 features)	LR	87.44 ± 3.37/86.79 ± 3.63	87.53 ± 7.96/88.58 ± 7.80	86.92 ± 4.40/83.59 ± 10.75	91.59 ± 1.77/90.17 ± 5.02	89.24 ± 3.73/88.98 ± 3.60
	XGB	86.17 ± 1.47/86.81 ± 3.58	91.79 ± 3.98/90.84 ± 6.62	77.44 ± 3.07/80.77 ± 7.83	86.45 ± 1.57/88.29 ± 3.08	88.97 ± 1.47/89.30 ± 3.08

A/B, A uses manually corrected segmentation labels and B uses a computer-assisted segmentation mask implemented by GLISTRboost but not manually corrected.

**Table 4:** Performance metrics of machine learning models for classifying short-term from long-term survival.

Feature (A/B)	Model	*p*-Value	HR	CI	C-Index	Brier Score
	RF	0.018/0.007	2.21/2.48	1.13–4.35/1.26–4.88	0.689/0.690	0.111/0.121
LASSO features	SVM	0.002/0.189	3.56/1.60	1.54–8.27/0.79–3.23	0.609/0.567	0.142/0.185
(1417/3857 features)	LR	0.062/0.099	1.88/1.76	0.96–3.71/0.89–3.46	0.636/0.623	0.211/0.144
	XGB	0.002/0.013	2.85/2.30	1.44–5.64/1.17–4.55	0.664/0.689	0.111/0.079
	RF	0.012/0.005	2.33/2.57	1.18–4.59/1.30–5.06	0.681/0.718	0.108/0.119
ALL features	SVM	0.002/0.189	3.56/1.60	1.54–8.27/0.79–3.23	0.610/0.567	0.145/0.184
(3861/3861 features)	LR	0.062/0.099	1.88/1.76	0.96–3.71/0.89–3.46	0.634/0.612	0.214/0.137
	XGB	0.037/0.013	2.08/2.30	1.03–4.18/1.17–4.55	0.652/0.689	0.131/0.080
	RF	0.0003/0.0003	3.24/3.24	1.63–4.43/1.63–4.43	0.719/0.719	0.113/0.113
ICC features	SVM	0.011/0.017	2.53/2.32	1.20–5.30/1.14–4.72	0.592/0.592	0.200/0.189
(1508/1508 features)	LR	0.002/0.002	2.85/2.85	1.44–5.64/1.44–5.64	0.695/0.695	0.173/0.173
	XGB	0.015/0.015	2.29/2.29	1.15–4.53/1.15–4.53	0.669/0.669	0.085/0.085
	RF	0.002/0.003	2.83/2.67	1.43–5.59/1.35–5.26	0.701/0.711	0.105/0.119
Combined ICC and LASSO	SVM	0.002/0.189	3.56/1.60	1.54–8.27/0.79–3.23	0.610/0.567	0.143/0.181
(2537/3858 features)	LR	0.062/0.140	1.88/1.66	0.96–3.71/0.84–3.27	0.634/0.623	0.212/0.164
	XGB	0.005/0.034	2.57/2.06	1.29–5.11/1.04–4.08	0.651/0.669	0.132/0.073

A/B, A uses manually corrected segmentation labels and B uses a computer-assisted segmentation mask implemented by GLISTRboost but not manually corrected.

## Data Availability

The original contributions presented in the study are included in the article, further inquiries can be directed to the corresponding author.
